# Action and learning shape the activity of neuronal circuits in the visual cortex

**DOI:** 10.1016/j.conb.2018.04.020

**Published:** 2018-10

**Authors:** Janelle MP Pakan, Valerio Francioni, Nathalie L Rochefort

**Affiliations:** 1 Center for Behavioral Brain Sciences, Institute of Cognitive Neurology and Dementia Research, Otto-von-Guericke University, Magdeburg, Germany; 2 German Center for Neurodegenerative Diseases, Magdeburg, Germany; 3 Centre for Discovery Brain Sciences, School of Biomedical Sciences, Edinburgh, United Kingdom; 4 Simons Initiative for the Developing Brain, Edinburgh, United Kingdom

## Abstract

Nonsensory variables strongly influence neuronal activity in the adult mouse primary visual cortex. Neuronal responses to visual stimuli are modulated by behavioural state, such as arousal and motor activity, and are shaped by experience. This dynamic process leads to neural representations in the visual cortex that reflect stimulus familiarity, expectations of reward and object location, and mismatch between self-motion and visual-flow. The recent development of genetic tools and recording techniques in awake behaving mice has enabled the investigation of the circuit mechanisms underlying state-dependent and experience-dependent neuronal representations in primary visual cortex. These neuronal circuits involve neuromodulatory, top-down cortico-cortical and thalamocortical pathways. The functions of nonsensory signals at this early stage of visual information processing are now beginning to be unravelled.

## Highlights

• Arousal and locomotion modulate neuronal activity in primary visual cortex.
• Neurons in primary visual cortex respond to visuomotor mismatch.
• Experience shapes neuronal responses to familiar stimuli, reward and object location.
• Neuronal representations of visual stimuli are modulated according to the behavioural relevance of the stimuli.
• Neuromodulatory, top-down and thalamocortical inputs convey arousal-related and motor-related signals to primary visual cortex.

**Current Opinion in Neurobiology** 2018, **52**:88–97

This review comes from a themed issue on **Systems neuroscience**

Edited by **Michael Long and Rosa Cossart**

For a complete overview see the Issue and the Editorial

Available online 1st May 2018

**https://doi.org.10.1016/j.conb.2018.04.020**

0959-4388/© 2018 The Authors. Published by Elsevier Ltd. This is an open access article under the CC BY license (http://creativecommons.org/licenses/by/4.0/).

## Introduction

The brain integrates sensory information to generate sensations, thoughts and motor actions that are relevant for an animal's behaviour. This process involves the integration of sensory inputs with internal information about the animal's behavioural-state and its previous experience. For the same sensory stimulus, the outcome may be very different: for example, the vision of a dog can either trigger an affectionate or a fearful reaction depending on our past experience and physical condition.

Historically, primary sensory areas were thought to function as feature detectors. These areas create a representation of the external world that would be transmitted to higher cortical areas where this representation would be integrated with information related to past experience and behavioural state. This view was supported by *in vivo* electrophysiological recordings performed in anaesthetised and often paralysed animals, which have revealed fundamental principles of neuronal encoding of visual features in the visual cortex [1, 2, 3]. However, the use of anaesthetised animals prevented the investigation of the influence of nonsensory variables on V1 neuronal activity. In addition, anaesthesia itself modifies neuronal activity [4].

In this review, we present recent experimental approaches that were developed to investigate neuronal representations in the cortex of awake behaving mice. We also review current knowledge about the integration of nonsensory information in the adult rodent primary visual cortex, describing the impact of state-dependent changes (arousal and locomotion) as well as past experience on V1 neuronal activity. We review identified pathways that provide state-dependent and motor-related information to V1 neurons. We then discuss current challenges in the standardization of experimental conditions for awake-behaving animals as well as issues raised by big data analyses. Finally, we discuss potential functions of these nonsensory signals at this early stage of visual information processing.

### Investigation of visual information processing in awake behaving mice

**Figure 1:**
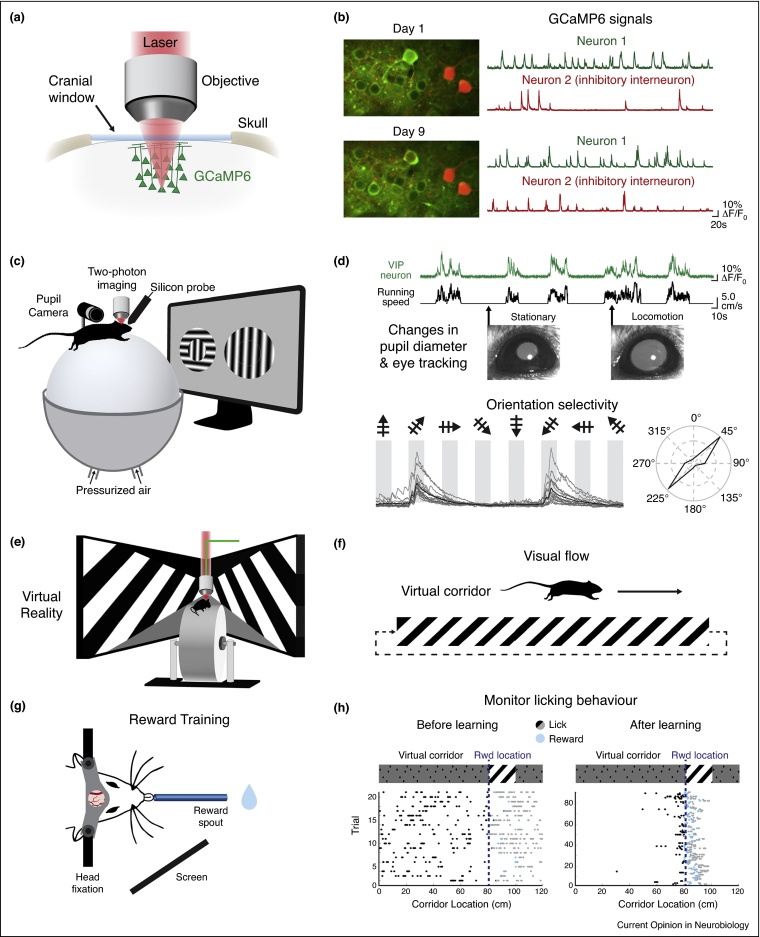
Experimental procedures for recording neuronal activity and correlated behavioural parameters in head-fixed awake behaving rodents. **(a)** Schematic of a cranial window preparation above primary visual cortex (V1) that allows chronic two-photon imaging of neurons labelled with a genetically encoded calcium indicator (GCaMP6). **(b)** GCaMP6-labelled neurons (green) can be imaged over multiple days and even weeks (example field of view from imaging Day 1 and 9). Relative changes in fluorescence (Δ*F*/*F*_0_) over time are used as a proxy read-out of neuronal activity. Signals from genetically defined subpopulations of cells can be isolated via a second fluorescent marker (e.g. tdTomato expression in somatostatin expressing inhibitory interneurons, shown here in red). **(c)** Head-fixed rodents can freely move on an air-supported styrofoam ball that acts as a spherical treadmill while calcium imaging and/or electrophysiological recordings are performed. Optical computer mice are used to assess running speed. Pupil diameter and eye-tracking can be recorded with cameras. In this configuration, animals can navigate an open virtual reality environment or view a visual stimulus where left/right behavioural choices can be made with motor movements. **(d)** Neuronal activity can be correlated with running speed and changes in pupil diameter, used as a measure of arousal. GCaMP6 signal from a V1 VIP expressing interneuron is shown in green and animal's speed in black. As traditionally done in anaesthetised animals, neuronal activity in V1 can be correlated with visual stimulation in the form of passively viewed stimuli, such as drifting gratings displayed on a screen. Bottom panel shows single trials (grey) and average response (black) of a GCaMP6-labelled neuron to different oriented gratings. Polar plot shows the amplitude of calcium transients in response to each orientation, normalised to the maximum response. **(e)** Head-fixed rodents can be placed in a virtual environment: animals run as if on a linear treadmill and, with surrounding screens, can navigate virtual corridors with defined wall patterns. Note that the spherical treadmill can be replaced by a cylindrical wheel where an optical encoder attached to the central axle records speed. **(f)** Using a virtual reality environment, the visual flow experienced by the animal can be measured and manipulated. An animal navigating along a virtual corridor creates visual flow: the experimenter can manipulate this coupling to create a mismatch between the visual flow and the animal's movement. **(g)** Experience-dependent neuronal changes in V1 can be studied using head-fixed animals learning visually guided tasks. Water deprived animals learn to lick during key experimental cues and goal-directed behaviours in order to receive water rewards through a spout. **(h)** Licking behaviour is monitored by a lick sensor on the spout during task performance. Example of a task in which the animal must lick at a certain reward location demarcated by a visual cue (oriented grating) along a virtual track. Each dot represents a lick: prereward licking (black dots), rewarded-licking at the right visual cue (blue dots) and postreward licks (grey dots). With learning, licking behaviour becomes tightly coupled to the location of the rewarded visual cue along the track.

The development of recording methods in awake behaving animals has led to seminal discoveries about the impact of action and learning on the activity of visual neurons in cats and nonhuman primates [5, 6, 7]. However, the lack of genetic tools in these species limits the investigation of the neuronal circuits underlying these experience-dependent changes in V1 neuronal activity. Such mechanisms can be studied in the mouse visual system by combining genetic tools and recordings in awake behaving mice. Using either electrophysiological recordings or two-photon imaging in head-fixed awake mice, it is now possible to monitor the activity of hundreds to thousands of neurons, during several days and weeks (Figure 1a–c). Neuronal activity is then correlated with both visual stimuli presented during the recordings as well as measured behavioural parameters such as task performance (Figure 1c,d). This approach not only reveals the impact of internal state and past experience on neuronal activity but also the dynamics of visual neuronal circuits during learning. Additionally, the availability of transgenic mice expressing Cre-recombinase in specific subtypes of inhibitory neurons has enabled the characterization of both excitatory and inhibitory activity in the visual cortex (Figure 1b).

### Arousal and locomotion increase the gain and modulate selectivity of visual responses

**Figure 2:**
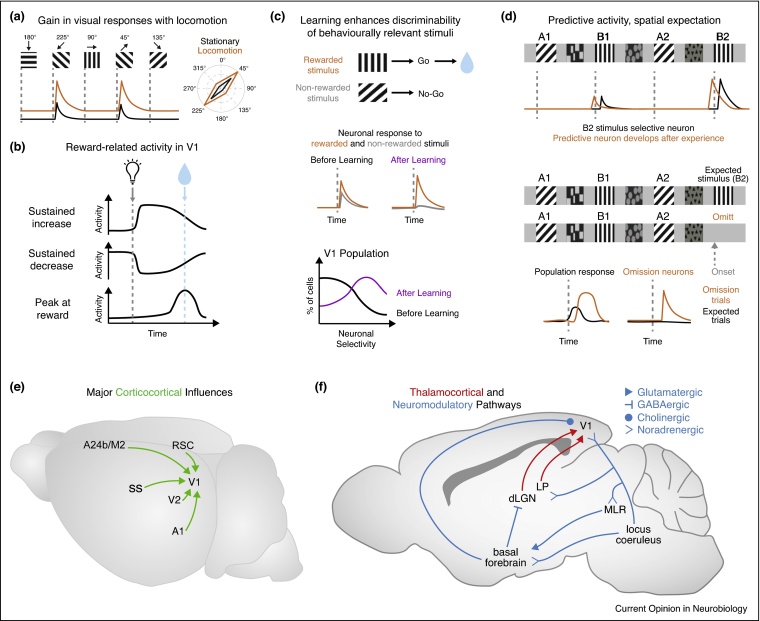
Neuronal activity in V1 is shaped by behavioural-state and experience-dependent processes, mediated through the integration of nonvisual inputs. **(a)** Schematic representing the increased gain in neuronal responses to oriented gratings during locomotion (orange trace) versus stationary periods (black trace). Both additive (as illustrated) and multiplicative gain modulations were reported in V1 excitatory neurons [22^•^]. Illustration is based on Ref. [10]. **(b)** Schematic of representations of reward timing in V1. After the learning of a task associating a visual cue (light bulb) and a reward (blue drop), neuronal responses in V1 predict the timing of reward events by sustaining either an increase or decrease in activity after a visual stimulus onset (grey dotted line) or peaking at the expected reward time (blue dotted line). Illustration is based on Refs. [37, 38]. **(c)** Schematic of V1 responses during the learning of a visually guided task. Example of a go/no-go task with two oriented gratings and only one grating is rewarded (blue drop). A schematic of the responses of a single neuron to the two presented stimuli show that neuronal discriminability between the rewarded (orange) and nonrewarded (grey) stimuli increases with task learning. On the population level (bottom panel), a higher proportion of neurons in V1 show increased selectivity to task-relevant gratings after learning (purple). Illustrations are based on Ref. [43]. **(d)** Schematic of responses to spatial expectation of visual stimuli in V1. Top panel: schematic of a paradigm where animals are presented with a sequence of visual cues along a virtual track. Traces illustrate neuronal responses to visual cues, before (black) and after (orange) repeated exposure to the same sequence. A population of V1 neurons show specific responses to a given visual stimulus (e.g. vertical grating) but also specific responses for a given stimulus at a particular spatial location (response to vertical grating at B2 location larger than B1). With experience, a population of neurons develop predictive responses, shifting the onset of their response to before the appearance of their preferred stimulus (orange trace). Bottom panel illustrates the effect of omitting an expected stimulus in a trained sequence. On the population level, when the stimulus is present there is an evoked response to the stimulus (black trace), but when the stimulus is un-expectantly omitted (orange trace), there is a large and delayed increase in activity. A subpopulation of neurons respond selectively to these omission events, and not to the initially expected stimulus. Illustration is based on Ref. [28^•^]. **(e)** Schematic of the major cortico-cortical inputs to V1, including top-down influences from higher visual areas (V2), the retrosplenial cortex (RSC) and secondary motor regions (A24b/M2) as well as inputs from other sensory modalities such as the primary auditory cortex (A1) and the somatosensory cortex (SS). Inputs from higher visual areas (V2) include connections from lateral, medial and mediolateral secondary visual areas. **(f)** Schematic of neuromodulatory and thalamocortical inputs to V1 that have been shown to influence V1 activity in awake behaving mice. LP, Lateral posterior nucleus; dLGN, dorsal lateral geniculate nucleus; MLR, mesencephalic locomotor region.

The impact of arousal and locomotion on the activity of visual cortex neurons has been most commonly studied using recordings in head-fixed rodents that are freely running on a ball [8] or a disk (Figure 1c,e). The running speed of the animal, pupil dilation and local field potentials (LFP) are used to assess state-dependent changes in awake rodents [9] (Figure 1d). An increase in arousal measured by an increase in running speed, or pupil dilation, correlates with an increase in the visually evoked activity of neurons in V1 [10, 11, 12, 13, 14•, 15, 16, 17••] and higher visual areas [18, 19]. This gain in the visual responses during locomotion has been shown to preserve the orientation preference and spatial frequency of excitatory neurons [10, 20•] (Figure 2a). During periods of high arousal while the animal is stationary, corresponding to periods of pupil dilation, responses at preferred orientations are enhanced, resulting in a sharpening of orientation selectivity [15]. During locomotion, neurons preferring high spatial frequencies display larger gain in visual responses than other neurons, suggesting an increased spatial resolution during locomotion [20^•^]. In addition, a shift towards higher temporal frequency preferences was observed in V1 and higher visual areas [18]. Finally, locomotion also increases spatial summation of V1 neurons by reducing surround suppression [11, 21].

Altogether, these results suggest a more robust and accurate encoding of visual stimuli during high arousal and locomotion. Concordantly, decoders used to infer the identity of the presented visual stimulus from the activity of V1 neurons, perform better when using activity during locomotion than during still periods [20•, 22•]. One potential mechanism underlying this increased stimulus discriminability is the decreased response variability both at the level of subthreshold membrane potentials [13, 14•] and noise correlations in neuronal populations [11, 16, 22•] during periods of locomotion and high arousal. In addition, it has been suggested that different cortical layers may encode visuo-locomotor information in different ways [11, 22•]. For instance, in layer 2/3, stimulus discriminability by decoders was improved mainly because of an increase in firing rate during locomotion. However, in layer 5, the increased stimulus discriminability was mainly the consequence of a decrease in noise correlations across the population [22^•^].

### Experience shapes the neuronal representation of visual stimuli in V1

#### Integrating self-motion with visual inputs: visual flow predictions and spatial expectations in V1

A fundamental function of the visual system is to enable the detection of moving stimuli and to navigate through the environment. For this, it is necessary to assess the relation between self-motion and the position and speed of a visual stimulus. This process requires an estimate of the animal's self-motion and a comparison of this estimation with visual inputs. The use of virtual reality is well suited to investigate the circuit mechanisms underlying this process since it allows measuring the speed and manipulating the visual flow experienced by the animal [23, 24] (Figure 1e,f).

As described above, in addition to feedforward visual inputs, motor-related signals modulate V1 activity. A subpopulation of neurons was found to detect mismatch between the animal's movement and the visual flow [12, 25]. These neurons have restricted receptive fields and thus respond to local mismatch in a specific portion of visual space, as is the case when an object moves into the visual space of a behaving animal [25]. These mismatch responses are shaped by active visuomotor experience, that is, by the experience of a given relation between the animal's own movement and the motion of a visual stimulus, both during development and in adulthood [26, 27••]. These results are consistent with a predictive coding interpretation of visual processing: since during normal development, the relation between visual-flow and self-movement is very consistent, this relation is thus predictable. This prediction could then be compared to feed-forward visual inputs in order to detect mismatches between the prediction and the visual stimuli: such mismatches would, for example, occur for objects moving independently of the animal.

Internal representations of the animal's self-movement are crucial for navigation and spatial expectations of visual stimuli. It was shown that a subset of V1 neurons respond specifically to a given stimulus placed in one location and less to the same stimulus at another location along a virtual track [28^•^] (Figure 2d). These responses were shaped by experience and became predictive: neurons responded before the expected encounter of the visual stimulus and to the absence of the expected stimulus (omission signal) [28^•^] (Figure 2d). These results are also consistent with predictive coding in the visual cortex: an internal representation of the visual scene is compared to feed-forward visual inputs, leading to experience-dependent representations of spatial expectations of visual stimuli.

#### Passive exposure either enhances or decreases responses to the exposed stimulus

Several studies have shown that the daily presentation of visual stimuli over consecutive days, without any associated reward or aversive stimuli, modifies the representation of these stimuli in V1. Electrophysiological recordings performed in awake head-fixed mice, placed in a tube, have revealed a long lasting (across weeks) stimulus-specific potentiation of visually evoked potentials in V1 layer 4 [29, 30]. This stimulus-specific response potentiation correlated with the habituation of a behavioural response. Initially the mice would respond to novel gratings with fidget-like movements of the forepaws, but these movements habituated in a stimulus-specific manner and over the same time course as the potentiation of V1 responses [30]. Another study using intrinsic signal imaging and chronic two-photon calcium imaging in anaesthetised animals also showed a stimulus-specific increase in the responses of layer 2/3 neurons to a daily presented stimulus [31]. However, this effect was only observed in mice that had been running for a cumulative time of at least one hour during the stimulus presentations across days [31]. These results are contrasted by a third study using the same experimental approach in awake running mice but showing a stimulus-specific decrease in the number of layer 2/3 visually responsive neurons across days [32]. The discrepancies between these studies highlight the challenges of standardizing experimental conditions and data analysis for high-throughput recordings in awake behaving mice (see last section of this review).

A potential source of variability could be that different neuronal subnetworks may display either stable or plastic neuronal responses during passive viewing. A recent study showed that 2 weeks after the presentation of high-contrast gratings of different sizes, a systematic shift towards smaller size preferences and greater surround suppression was observed in a subpopulation of weakly responsive neurons, while the majority of excitatory neurons maintained stable responses [33].

Finally, another form of experience-dependent changes in V1 activity during passive exposure was demonstrated after repeated exposure to rapid sequences of stimuli [34] and fast-moving spots [35]. Such protocols triggered a recall of neuronal activity when some stimuli were omitted from the sequence. This effect was strongly dependent on the timing of the sequence [34] and was observed in anaesthetised animals and quiet, immobile awake animals. However, this effect was not observed in awake mice with facial/whisker movement and irregular, high-frequency LFP activity, which are characteristic of behaving animals [35].

#### Learning a behavioural task correlates with enhanced neuronal representations of relevant stimuli

By using either rewards or aversive stimuli, mice engage in performing and learning a task. Most commonly, animals are deprived of water or food and a given visual stimulus is paired with either reward [36] (Figure 1g,h).

Neurons in deep layers of rat V1 were shown to acquire responses specific to the timing interval preceding a reward [37] (Figure 2b). For this task, light flashes were delivered to either the left or the right eye; depending on which eye was stimulated, the animal had to perform a different number of licks (i.e. over time) to obtain a water-reward. The acquisition, but not the expression, of this reward-timing activity has since been shown to depend on cholinergic inputs from basal forebrain projections to V1 [38, 39].

Rodents can also learn to discriminate a rewarded visual stimulus from a nonrewarded one [40, 41, 42] (Figure 1g,h). Combining chronic two-photon calcium imaging in V1 layer 2/3 neurons with a go/no-go discrimination task in a virtual reality environment, a study has revealed that the V1 population becomes better at discriminating the rewarded from the nonrewarded stimulus [43] (Figure 2c). Task-specific changes in the activity of layer 2/3 neurons have also been observed in aversive conditioning tasks, in which, for example, mice had to detect a visual stimulus by initiating running on a treadmill; failure to run triggered a mild tail shock [32]. In this study, learning modulated the activity of both excitatory and somatostatin (SST)-expressing inhibitory neurons. Additionally, by imaging changes in fluorescence of GCaMP6-labelled axons from the retrosplenial cortex in V1, this study also showed an increase of calcium transients in these top-down inputs during learning [32].

The relationship between V1 encoding of relevant stimuli and behavioural performance was also recently investigated in a go/no-go visual stimulus detection task combined with *in vivo* two-photon imaging of OGB-labelled neurons [44]. The results showed that visual detection of a rewarded stimulus strongly correlates with the timing accuracy and sequence in which clusters of layer 2/3 neurons are active [44, 45]. Using classical conditioning and electrophysiological recordings in adult mice, another study has shown that learning of orientation discrimination under classical conditioning correlated with increased neuronal discriminability between the rewarded and unrewarded stimulus, greater orientation tuning and improved contrast sensitivity. Notably, the improved representation of the relevant stimulus in V1 was fully developed before any improvement in the animal's behavioural performance [46^•^].

Finally, a direct test of the impact of stimulus behavioural relevance was performed by assessing contrast adaptation responses (reduced responses to sustained stimuli) of V1 neurons in a visually guided task in a virtual environment. While layer 2/3 neurons display an adaptation of their response for stimuli that were not relevant for the task, such adaptation was absent when stimuli became relevant to solve the same task [47^••^].

Altogether, these results support the view of a dynamic regulation of visual information processing in V1 based on the behavioural relevance of the stimulus. In this way, behaviourally salient visual representations are enhanced and stabilised while responses to nonrelevant stimuli are suppressed, for example, through mechanisms of adaptation. The long-term stability of these representations may also depend on their behavioural relevance [48].

### Neuronal circuits underlying the integration of nonsensory inputs in the primary visual cortex

The precise origin of nonsensory inputs to V1 is still unclear. Recent advances in circuit mapping techniques (such as MAPseq technologies [49]) are bringing researchers closer to identifying these anatomical pathways on a whole brain scale. However, these large datasets now need to be integrated with functional recordings from neurons. So far, a number of studies have indicated that nonsensory signals can be conveyed through top-down cortico-cortical and thalamic inputs to V1, as well as via neuromodulatory pathways.

Neuromodulation has been shown to be a key mediator of brain state changes [9]. Both cholinergic [50] and noradrenergic [14^•^] inputs were shown to drive locomotion-related gain changes in V1. Results from stimulation of cholinergic neurons in basal forebrain [51] or stimulation of afferent projections to the basal forebrain [52], as well as calcium imaging of cholinergic projections in V1 [15], are all consistent with a role of cholinergic inputs in gain modulation of V1 activity [53]. It is also likely that other neuromodulators, including serotonin (5-HT), are involved in modulating V1 circuit activity during behaviour.

Changes in arousal or locomotion do not only modulate the activity of excitatory neurons but also the activity of inhibitory ones, including the three nonoverlapping classes of vasoactive intestinal peptide (VIP)-, SST- and parvalbumin-expressing neurons [14•, 15, 17••, 26, 50, 54, 55]. One proposed mechanism for gain modulation during locomotion has been an activation of VIP neurons through nicotinic acetylcholine receptors, leading to an inhibition of SST interneurons and a disinhibition of excitatory neurons [50]. Consistent with this mechanism, VIP neuronal activity reliably increases during periods of arousal and locomotion [17••, 50, 54] (Figure 1d) and activation of VIP neurons in V1 was found to elicit responses with similar properties as those elicited during locomotion [50, 56].

However, SST interneurons were shown to be strongly responsive to visual stimuli and to further increase their activity during locomotion [17••, 26, 54, 55], challenging the generality of the disinhibition model. In addition, the amplitude of this increased gain varies depending on the size of the stimulus and screen illumination [54] and SST neuronal activity is minimally, or slightly negatively, modulated by locomotion in darkness [17••, 26, 50, 54]. This context-dependent change in SST responses was recently described in a computational model as an emerging property of circuits that include synaptic interactions between diverse neuronal populations and a nonlinear input–output relationship for each population [57^•^]. Considering that, in the rodent cortex, both excitatory and inhibitory neurons have different types of receptors (nicotinic and muscarinic) for different neuromodulators [14•, 51, 52, 53, 58, 59, 60, 61], differential recruitment of interneuron subtypes may be triggered through a timely, coordinated release of different neuromodulators, depending on the behavioural context of the animal. Another hypothesis is that arousal and locomotion not only elicit the release of neuromodulators but also trigger direct excitatory inputs to V1.

Motor-related inputs to V1 are conveyed by axonal projections from a subdivision of anterior cingulate cortex (A24b) and an adjacent part of secondary motor cortex (M2) [27^••^]. These excitatory inputs were shown to drive motor and mismatch signals in V1 and adapt in an experience-dependent manner [27^••^]. Both layer 2/3 excitatory mismatch neurons and a subset of VIP interneurons were found to receive motor-related excitatory input, while a subset of SST interneurons was more strongly activated by visual flow [26]. A proposed mechanism is thus that mismatch excitatory neurons compute the difference between an inhibitory visual input provided by a subset of SST neurons, and an excitatory prediction of visual input based on motor output and provided by top-down connections [26, 27••].

Finally, the integration of motor-related or experience-related information with visual inputs could occur in subcortical nuclei and then be transmitted to cortical areas. Locomotion-related activity was observed in thalamic nuclei processing visual information, the dorsal lateral geniculate nucleus (dLGN) [11] and the lateral posterior nucleus [62]. The contribution of feedback cortico-thalamic inputs to these activities remains unclear. Running speed also correlates with narrowband gamma power oscillations in V1 [52, 63]. These oscillations were also found in the dLGN and silencing V1 optogenetically did not abolish them, suggesting that narrowband gamma power in V1 is mediated primarily by the rhythmic firing of LGN neurons [63].

Many aspects of the mechanisms underlying the impact of action and learning on V1 neuronal circuit activity remain unknown. These mechanisms involve diverse interconnected pathways: cortico-cortical, cortico-thalamo-cortical and neuromodulatory circuits (Figure 2e,f; see also Figure 1 of [27^••^]). In addition to the inputs described in this review, inputs from other sensory areas, in particular from the auditory cortex, also modulate V1 neuronal activity [64, 65]. Each of these pathways can act independently or in synergy with each other, on different time and spatial scales and at different levels of neuronal activity, from membrane potential dynamics, to spike rates and neuronal population coding. Future challenges include the development of analysis tools that could encompass this high number of variables at different time scales. A comparison of neuronal activities across different sensory areas may reveal common circuit mechanisms; a recent study has combined large-scale imaging and voltage-sensitive dyes to demonstrate similar effects of arousal on neuronal responses to trains of sensory stimuli in visual, auditory and somatosensory areas [66].

### Challenges: standardization of experimental conditions and analysis methods

In recent years, the number of neurons in which activity can be measured over long periods of time in awake behaving mice has increased exponentially. Such experiments provide extremely rich datasets including changes in neuronal population activity as well as different behavioural parameters measured over time. A current challenge is the development of standardised analysis tools for the reliable extraction of spiking activity [67] or calcium transients [68, 69, 70, 71] of individual neurons from these large datasets. In addition, the criteria for the inclusion or exclusion of recorded neurons in the analysis strongly vary across studies making direct comparisons difficult. One example of such variability of results is the reported proportion of neurons in V1 whose activity is modulated by locomotion: this proportion ranges from 26% [11] to 55% [20^•^], 78% [22^•^] and 89% [33], in different studies. These challenges apply not only to studies of the visual system but also more broadly to *in vivo* calcium imaging datasets. Sharing custom-developed software that was used for data analysis in each calcium imaging publication would help increasing reproducibility for studies investigating visual processing, but also for a more accurate comparison of neuronal activity across cortical regions [72].

Another potential source of variability comes from the experimental conditions: housing conditions [73], noise and light levels in the imaging set-up, and circadian phase of the animals [74], were all found to affect the activity of visual neurons. A standardised quantification of arousal can be achieved by measuring LFP, pupil dilation and movement [9]. A detailed tracking and segmentation of the different components of a given behaviour [75] would also help to reduce variability across experiments and laboratories.

Finally, the development of methods to record neuronal activity and to track the behaviour of freely moving animals [76] would enable the study of more evolutionary relevant behaviours [77, 78] than the ones tested in head-fixed mice. The influence of nonvisual inputs on visual processing may indeed vary between innate, natural behaviours that encompass multisensory and behaviourally relevant inputs and controlled behavioural tasks performed in front of a screen. Lastly, visual information processing may differ between the smaller but highly connected mouse brain and the highly hierarchical primate brain [5, 79, 80], with nonsensory influences in rodents being more prominent in primary sensory regions.

## Conclusion

Recent studies in awake mice have revealed a previously unexpected diversity and plasticity of neuronal representations in the adult visual system. While the studies presented in this review show that action and learning shape neuronal representations, very little is known about the behavioural advantage or benefit of these activity-dependent and experience-dependent representations in the visual system. Potential functions include improvements in the perception of stationary and moving objects, object recognition, movement control, spatial navigation, and acquisition of visually guided actions. The experimental challenge is to establish a causal link between specific neuronal representations and behavioural output.

One study has shown that locomotion does correlate with a significant improvement in the detection of low-contrast and medium-contrast gratings [13]: this decrease in perceptual threshold correlates with the increased signal-to-noise ratio of V1 visual responses during locomotion. Another study suggests a role for V1 activity in the acquisition of a visually guided task [46^•^]. Among other potential functions, prediction of visual flow based on self-movement could be used to detect mismatch between this prediction and moving objects, enhancing the detection of objects that move independently from the animal. Combined with the integration of running speed information [24], spatial expectations may help navigation by providing spatial landmarks. Lastly, since V1 neurons project to the superior colliculus and to brainstem nuclei via cortico-fugal projections, extravisual inputs to V1 neurons may directly be involved in the modulation of innate motor behaviours such as experience-dependent optokinetic reflex potentiation [81] or light-induced arrest behaviour [82].

## Conflict of interest statement

Nothing declared.

## References and recommended reading

Papers of particular interest, published within the period of review, have been highlighted as:

• of special interest
•• of outstanding interest
